# Novel Glycomimetics Protect against Glycated Low-Density Lipoprotein-Induced Vascular Calcification In Vitro via Attenuation of the RAGE/ERK/CREB Pathway

**DOI:** 10.3390/cells13040312

**Published:** 2024-02-08

**Authors:** Gary P. Sidgwick, Ria Weston, Ayman M. Mahmoud, Andrew Schiro, Ferdinand Serracino-Inglott, Shikha M. Tandel, Sarah Skeoch, Ian N. Bruce, Alan M. Jones, M. Yvonne Alexander, Fiona L. Wilkinson

**Affiliations:** 1 Department of Life Sciences, Manchester Metropolitan University, Manchester M1 5GD, UKr.weston@mmu.ac.uk (R.W.); a.mahmoud@mmu.ac.uk (A.M.M.); ferdinand.serracino-inglott@mft.nhs.uk (F.S.-I.); standel01@qub.ac.uk (S.M.T.); a.m.jones.2@bham.ac.uk (A.M.J.); y.alexander@mmu.ac.uk (M.Y.A.); 2 Cardiovascular Research Institute, University of Manchester, Manchester M13 9PL, UK; druschiro@hotmail.com; 3 Vascular Unit, Manchester University Hospitals NHS Foundation Trust, Manchester M13 9WL, UK; 4 Centre for Epidemiology Versus Arthritis, University of Manchester, Manchester M13 9PL, UK; sarah.skeoch1@nhs.net (S.S.); ian.bruce@manchester.ac.uk (I.N.B.); 5 National Institute for Health Research Manchester Biomedical Research Centre, Manchester University Hospitals NHS Foundation Trust, Manchester Academic Health Science Centre, Manchester M13 9PL, UK; 6 Royal National Hospital for Rheumatic Diseases, Bath BA1 1RL, UK; 7 School of Pharmacy, University of Birmingham, Birmingham B15 2TT, UK

**Keywords:** vascular calcification, glycation, heparan sulphate mimetics, critical limb ischemia

## Abstract

Heparan sulphate (HS) can act as a co-receptor on the cell surface and alterations in this process underpin many pathological conditions. We have previously described the usefulness of mimics of HS (glycomimetics) in protection against β-glycerophosphate-induced vascular calcification and in the restoration of the functional capacity of diabetic endothelial colony-forming cells in vitro. This study aims to investigate whether our novel glycomimetic compounds can attenuate glycated low-density lipoprotein (g-LDL)-induced calcification by inhibiting RAGE signalling within the context of critical limb ischemia (CLI). We used an established osteogenic in vitro vascular smooth muscle cell (VSMC) model. Osteoprotegerin (OPG), sclerostin and glycation levels were all significantly increased in CLI serum compared to healthy controls, while the vascular calcification marker osteocalcin (OCN) was down-regulated in CLI patients vs. controls. Incubation with both CLI serum and g-LDL (10 µg/mL) significantly increased VSMC calcification vs. controls after 21 days, with CLI serum-induced calcification apparent after only 10 days. Glycomimetics (C2 and C3) significantly inhibited g-LDL and CLI serum-induced mineralisation, as shown by a reduction in alizarin red (AR) staining and alkaline phosphatase (ALP) activity. Furthermore, secretion of the osteogenic marker OCN was significantly reduced in VSMCs incubated with CLI serum in the presence of glycomimetics. Phosphorylation of cyclic AMP response element-binding protein (CREB) was significantly increased in g-LDL-treated cells vs. untreated controls, which was attenuated with glycomimetics. Blocking CREB activation with a pharmacological inhibitor 666-15 replicated the protective effects of glycomimetics, evidenced by elevated AR staining. In silico molecular docking simulations revealed the binding affinity of the glycomimetics C2 and C3 with the V domain of RAGE. In conclusion, these findings demonstrate that novel glycomimetics, C2 and C3 have potent anti-calcification properties in vitro, inhibiting both g-LDL and CLI serum-induced VSMC mineralisation via the inhibition of LDLR, RAGE, CREB and subsequent expression of the downstream osteogenic markers, ALP and OCN.

## 1. Introduction

Significant advances have been made towards our understanding of vascular and resident stem cell biology and how vascular calcification reflects cellular plasticity within this niche [1,2]. Studies have shown a direct link between lower limb arterial calcification and cardiovascular events in patients with critical limb ischemia (CLI) [3,4]; although the role that calcification plays in plaque vulnerability is still unclear, it is still a major problem in the clinic. Vascular calcification is driven in part by dyslipidaemia and elevated levels of advanced glycation end products (AGEs) [5,6] coupled with its receptor (RAGE) [7,8], both of which act as an independent predictor of limb amputation [4] and premature death [9,10]. Indeed, low-density lipoprotein (LDL) itself is prone to glycation in this environment [11,12]. Therefore, anti-glycation activity and prevention of AGEs formation has become an attractive therapeutic target for improved management of calcification, potentially reducing the costly need for amputation in this patient group. However, the use of RAGE inhibitors has been trialled without much success, due to adverse side effects, reviewed by Prasad et al. [8], leaving the search for improved calcification blockers as a clinically unmet need.

The receptor for advanced glycation end products (RAGE) exists in a complex with heparan sulphate (HS) [13], a ubiquitous polysaccharide found covalently linked to membrane and matrix proteoglycans (PG) and known to promote reactive oxygen species (ROS) production [14] in addition to vascular calcification [15]. HS, a highly sulphated glycosaminoglycan, regulates numerous biological processes strengthening the structure and stability of the extracellular matrix (ECM) [16,17]. We have previously shown a correlation of impaired cell migration with structural alterations of HSPGs in an in vitro model of aging cells [18]. Our previous findings support the emerging concepts utilising glycomimetics as drugs where we have shown their beneficial potential for modulating signalling pathways, due to their effects on the mobilization and the potentiation of progenitor and stem cells from mesenchymal lineages [19].

We have previously identified and synthesised a promising series of small molecule HS mimics, which act in a protective manner against lipid-induced endothelial damage in vitro and ex vivo with the involvement of Akt/eNOS and Nrf2/ARE signalling [20], β-glycerophosphate (β-GP)-induced vascular smooth muscle cell (VSMC) calcification via c-met/notch3/hes1 signalling [21] and restore the functional capacity of diabetic endothelial colony forming cells in vitro [19]. Here, we investigated the mechanism of action of these novel HS-related compounds, (termed C2 and C3) in an in vitro glycated LDL (g-LDL)-induced osteogenic VSMC differentiation process, as a potential new strategy for attenuation of calcification.

## 2. Materials and Methods

### 2.1. Human Tissue and Serum Collection and Culture of Human VSMCs

Coronary artery VSMCs were purchased from Caltag Medsystems, (Buckingham, UK) and maintained using VSMC growth medium type 2 (PromoCell GmbH, Heidelberg, Germany), which was changed twice weekly. All cells were used between passages 4 and 8, with all experiments performed in duplicate or triplicate using three different populations of VSMCs. Informed consent was also obtained to harvest human tibial and popliteal arterial specimens (*n* = 25), following below-knee amputation in patients with critical limb ischemia (CLI; ethics reference 14/NW/1062), with arterial segments being processed for wax embedding and histopathological analysis [22]. Blood samples were also obtained from these patients, and the serum was harvested for analysis of markers of bone metabolism, glycation and the ability to induce calcification in VSMCs. Measurement of these markers was also undertaken in serum from healthy controls collected as part of another observational study and used for comparison (ethics reference 12/NW/0117). All procedures were in accordance with institutional guidelines and the Declaration of Helsinki.

### 2.2. Histopathological Analysis

Arterial sections (7 µm) were stained with alizarin red (AR) to distinguish areas of vascular calcification, as previously described [23]. Consecutive sections were stained with anti-RAGE antibody (Novus Biologicals, Colorado, USA; 5 µg/mL) or non-immune rabbit IgG control (Vector Laboratories, Newark, CA, USA; 5 µg/mL) overnight at 4 °C and developed using the biotin–peroxidase-3′,3′-diaminobenzidine (Vector Laboratories, California, USA) system followed by counterstaining with Harris haematoxylin, as previously described [23]. Sections were visualised using the Panoramic SCAN with Zeiss Plan-apochromat 20×/0.8 objective (3D Histotech/Laser2000, Ringstead, UK).

### 2.3. Bioplex Assay

The levels of bone marker-related proteins sclerostin (SOST), osteocalcin (OCN) and osteoprotegerin (OPG) in both CLI patient serum and healthy controls, and conditioned culture media were determined via a multiplex array analysis using a Milliplex Luminex xMAP™ bone marker panel (Merck Millipore, Burlington, MA, USA), performed as per manufacturer’s protocol.

### 2.4. Glycation Analysis

The extent of glycation was assessed using the 2,4,6-Trinitrobenzene Sulphonic Acid (TMBSA) assay (Sigma, Suffolk, UK) to quantify free versus glycated amino acid residues, as per manufacturer’s instructions, and absorbance was determined at 335 nm. Values were normalised based on protein concentration, as quantified by a bicinchoninic acid (BCA) assay (ThermoScientific, Waltham, MA, USA, as per manufacturer’s instructions) and expressed as the degree in reduction of absorbance of glycated-BSA (g-BSA) compared to a BSA standard curve (Supplementary Figure S1C) [24,25,26].

### 2.5. In Vitro Model of Calcification and Treatments

VSMCs were seeded in 6-well plates at 2 × 10^5^ cells/well and left to adhere for 24 h in DMEM (Lonza, Slough, UK) supplemented with 10% foetal bovine serum (FBS) (Gibco/Life Technologies, Inchinnan, UK), 100 U/mL penicillin, 100 mg/mL streptomycin, L-glutamine (Lonza, Slough, UK) and 2.6 mmol/l calcium chloride (CaCl_2_; Sigma, Suffolk, UK). Respective test treatments were added for up to 28 days depending on experiments performed, with concentrations and time points as specified in the text and figure legends. Media were changed twice weekly, while control cells were treated with vehicle alone.

Serum isolated from CLI patients was used in the cell culture model at a concentration of 5% with the addition of 5 mM β-GP being used as our reference calcification model, in order to establish the effect of g-LDL on the osteogenic differentiation of VSMCs (Supplementary Figure S1A,B).

Glycation of LDL was carried out by incubating 1 mg/mL of filtered sterile LDL (Alfa Aesar, Lancashire, UK) with 50 mM of methylglyoxal (Sigma, Suffolk, UK) for 7 days at 37 °C, followed by dialysis against PBS (pH 7.4) at 4 °C with 3 buffer changes over 24 h at 4 °C, using slide-a-lyser cassettes (2000 MW cut-off, Thermo Scientific, Altrincham, UK). The extent of glycation was quantified spectrophotometrically by determining the absorbance profile between 220 nm and 500 nm, and at 282 nm [27], and via the TNBSA assay as described previously (Supplementary Figure S1C). The g-LDL was stored at 4 °C and used within 7 days of preparation, at a concentration of 10 µg/mL. Novel glycomimetic compounds (C2 and C3) [20] were used to treat VSMCs at a concentration of 1 µM in complete media.

### 2.6. Alizarin Red (AR) Staining

The level of VSMC calcification was determined by AR staining. Cells were washed with PBS, fixed in 4% paraformaldehyde for 10 min, then washed and stained with 2% AR at pH 4.2 (Sigma, UK) for 5 min. Excess stain was washed off with deionised water three times over an hour, and cells were imaged using a 4x objective with a Zeiss Primovert inverted phase contrast microscope (Carl Zeiss Microscopy, Cambridge, UK). Calcification was quantified via elution of AR stain with 10% formic acid, with absorbance determined at 414 nm, as previously described [22]. For all experiments, representative images are shown. Data were expressed as fold change in absorbance compared to untreated controls.

### 2.7. Alkaline Phosphatase (ALP) Assay

Cells were seeded in 6-well plates at 2 × 10^5^ cells/well. Proteins were extracted at the required time points by freeze–thawing the cells in 0.05% Triton X-100 in PBS, and total protein content was measured using the BCA assay (Thermo Scientific, UK). Total protein (15 μg) was assayed for ALP activity by incubation with p-Nitrophenyl phosphate liquid substrate system (Sigma, UK) for 2 h at 37 °C, with absorbance measured at 405 nm, as previously described [28], and calculated as nmol/mL ρ-nitrophenol converted/μg of protein/min^−^^1^. The data were collected from three experiments performed in triplicate.

### 2.8. Western Blot Analysis and Phospho-Kinase Array

Levels of a range of proteins were determined using Western blotting and phosphor-kinase array analysis. In brief, cells were harvested and lysed using RIPA buffer (Millipore, Burlington, MA, USA) containing protease and phosphatase inhibitors (Sigma, UK), and total protein was quantified using the BCA protein assay kit (Thermo Scientific, UK). A human Proteome Profiler™ phospho-kinase array (R&D systems, Minneapolis, MN, USA) was performed, as per the manufacturer’s instructions, to identify targets of interest, followed by validation using Western blotting. Samples were loaded onto a 4–12% Bis/Tris gel (Life Technologies, UK), separated by electrophoresis at 200 V for 45 min, and then transferred onto a nitrocellulose membrane using wet transfer for 75 min at 35 V. The membrane was blocked with 5% skimmed milk/PBS-tween followed by incubation with the primary antibodies in blocking buffer on a shaker for one hour at room temperature or at 4 °C overnight. Following washing with PBS-tween, samples were incubated for 1 h with an appropriate HRP-conjugated secondary antibody (Dako, Agilent, Santa Clara, CA, USA), washed again and developed using ECL reagent (BioRad, Watford, UK) and imaged using a Biorad ChemiDoc™ Touch system. α-Tubulin (Abcam, Cambridge, UK; 1:5000) was used as a loading control. A full list of primary antibodies used in this study is shown in Supplementary Table S1.

### 2.9. In Silico Molecular Docking

The affinity of the glycomimetics (C2 and C3) towards human RAGE was explored using molecular docking. The crystal structure of human RAGE was obtained from the protein data bank (PDB: 3CJJ). The protein was prepared for docking through optimisation using Autodock Tools (ADT; v1.5.6), and PyRx virtual screening software (version 0.8) was employed for the execution of docking [29]. The binding affinity of the glycomimetics C2 and C3 and the RAGE inhibitor azeliragon (TTP488) with the V domain of RAGE to which AGEs bind [30], was investigated. PyMOL (v2.3.2) was employed for molecular visualisation and inspection of the binding modes, and protein–ligand interactions were obtained using LigPlot (v2.2.8) [31].

### 2.10. Statistical Analysis

All experiments were repeated in triplicate, with data quoted as mean +/− standard error of the mean (SEM). Normality of the data was tested using the Kolmogorov–Smirnov and the Shapiro–Wilk tests and statistical analysis was either performed using the Mann–Whitney U test or one-way ANOVA followed by Tukey’s test on GraphPad Prism 8 (GraphPad Software, La Jolla, CA, USA), as appropriate, with *p* < 0.05 considered significant. Correlations were analysed using Pearson’s correlation, with *p* < 0.05 considered significant.

## 3. Results

### 3.1. Patients with CLI Exhibit Extensive Arterial Vascular Calcification

**Figure 1:**
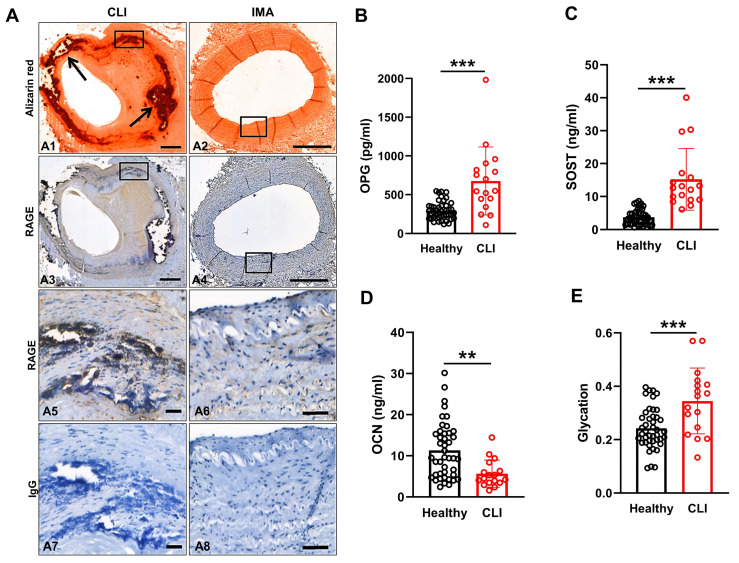
Vascular calcification in CLI patient peripheral arteries and blood. (**A**) Representative consecutive arterial sections from patients with chronic limb ischaemia (CLI) undergoing lower limb amputation and internal mammary artery (IMA) sections, taken from patients undergoing a coronary artery bypass graft surgery, which were stained with alizarin red to visualise regions of calcification (black arrows). RAGE immunoreactivity was detected at high levels in areas of calcification (black arrows) versus IMA where lower levels of RAGE were detected in smooth muscle and endothelial cells. Boxed areas in (**A1**–**A4**) are enlarged in (**A5**–**A8**). IgG controls were negative. Scale bars = 500 µm (**A1**–**A4**) and 50 µm (**A5**–**A8**). (**B**–**D**) Patient serum levels of bone marker proteins, using a Milliplex Luminex xMAP™ panel, and (**E**) glycated proteins. Serum from CLI patients (*n* = 17) and healthy controls (*n* = 43) were analysed. Data were analysed using a Mann–Whitney U test (** *p* < 0.01, *** *p* < 0.001).

Histopathological analysis was performed on popliteal arterial tissue isolated from patients with CLI at the time of surgical amputation (*n* = 17; Figure 1A), to characterise plaque morphology. To determine whether local RAGE expression was upregulated in calcified versus non-calcified arteries, sections of calcified popliteal artery and non-calcified internal mammary artery (IMA) were stained immunohistochemically with anti-human RAGE antibody and AR. Figure 1(A1) shows a representative section of a CLI patient’s popliteal arterial section, displaying the presence of calcification by AR staining, as well as positive immunolocalisation of RAGE within the vicinity of calcified areas of the vessel wall and a distinct lack of RAGE staining in non-calcified areas (Figure 1(A3,A5)). Vascular calcification was observed in all CLI patient tissue, compared to IMA samples from patients undergoing coronary artery bypass grafting, which do not exhibit atherosclerotic plaque, and have little or no evidence of calcification (Figure 1(A2)), which supports our previous findings [23] and those of others [32]. Furthermore, the extent of RAGE-positive immunoreactivity was highly prevalent in regions of calcification versus non-calcified areas of the same arterial segment (Figure 1(A3)). The presence of RAGE staining in the IMA was minimal and located predominantly along the endothelium (Figure 1(A4,A6)). All IgG controls were negative (Figure 1(A7,A8)).

### 3.2. CLI Patient Serum Exhibits Elevated Glycation and Bone-Related Proteins

Glycosylation plays a vital role in cardiovascular health [33] and induces VSMC calcification in vitro [34]. Furthermore, since Younis et al. have demonstrated that levels of g-LDL are raised in the plasma of hyperlipidaemic and hyperglycaemic patients compared to healthy controls [35,36], it was next of interest to ascertain whether the CLI patients had elevated circulatory levels of glycated proteins compared to controls (*n* = 43). In addition, to identify osteogenic factors that may be contributing to arterial vascular calcification and to the apparent localised up-regulated RAGE expression in this patient group, venous blood samples were harvested and serum analysed (*n* = 17) for biomarkers of calcification using a Milliplex Luminex xMAP™ (Merck Millipore, Watford, UK) panel.

Bone-related proteins, osteprotegerin (OPG; *p* < 0.001) and sclerostin (SOST; *p* < 0.001) were significantly elevated in the CLI patient serum when compared to healthy control serum (Figure 1B,C). However, osteocalcin (OCN; *p* < 0.01) was lower in CLI patient samples compared to controls (Figure 1D). As expected, the level of glycation observed in the CLI serum was significantly higher than in healthy controls (*p* < 0.001; Figure 1E).

### 3.3. Glycomimetics Block CLI Patient Serum-Induced Calcification of Human VSMCs In Vitro

**Figure 2:**
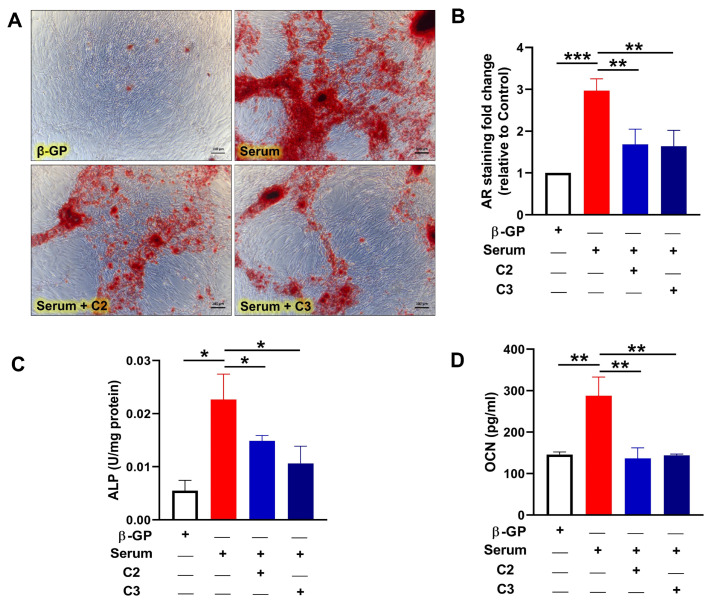
Inhibition of serum-induced calcification by novel glycomimetic compounds. VSMCs were treated with serum from patients with chronic limb ischemia (CLI; 5% patient serum/5% FBS + 5 mM β-GP), with and without novel glycomimetic compounds C2 and C3 (1 µM). Markers of calcification, alizarin red staining, alkaline phosphatase (ALP) activity and osteocalcin (OCN) levels were assessed and demonstrated that glycomimetics attenuate serum-induced calcification. (**A**) Alizarin red staining on day 10 highlights calcification (scale bar = 100 µm) which was (**B**) quantified and represented as fold change versus β-GP control. (**C**) ALP activity on day 4 following the addition of treatments. (**D**) Conditioned media was collected on day 7 and analysed using Bioplex array technology. Data are mean ± SEM, *n* = 3 independent experiments. * *p* < 0.05, ** *p* < 0.01 and *** *p* < 0.001.

Since CLI patient serum contained elevated glycation and osteogenic markers, we next investigated whether treatment with CLI serum would modulate the osteogenic differentiation of human VSMCs in an in vitro calcification model, and whether this could be attenuated by the addition of glycomimetics. VSMCs were incubated in osteogenic media supplemented with 5% CLI patient serum, in the presence or absence of glycomimetic C2 or C3, and the level of calcification was determined by quantifying AR staining. The serum-treated VSMCs showed an accelerated onset of mineralisation (*n* = 3), with a 2.9-fold increase in calcification evident after 10 days, compared with the osteogenic control conditions (*p* < 0.001; Figure 2A,B), considerably earlier than in our standard β-GP-induced calcification assays of 14–21 days [22,23]. This serum-induced differentiation was significantly inhibited in the presence of both C2 and C3 glycomimetics (*p* < 0.01; Figure 2A,B).

Further validation of the deposition of a mineralised matrix and its inhibition by glycomimetics was demonstrated by a significant elevation of an early marker of osteogenic differentiation, ALP activity, (*p* < 0.05), on day 4, in the presence of CLI serum versus osteogenic conditions (Figure 2C), which was markedly reduced in the presence of both C2 and C3 (*p* < 0.05). The levels of the later osteogenic marker, OCN were measured on day 7 and found to be significantly elevated in the cells incubated in the presence of CLI serum, compared to osteogenic controls (*p* < 0.01), yet significantly reduced when treated with C2 or C3, *p* < 0.01 (Figure 2D).

### 3.4. Glycomimetics Inhibit g-LDL-Induced Calcification of Human VSMCs In Vitro

**Figure 3:**
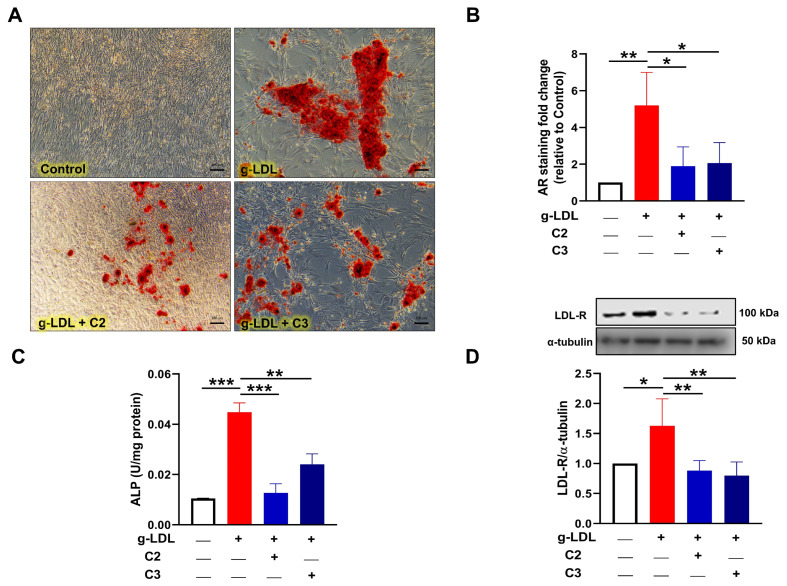
Glycated LDL-induced calcification is attenuated by novel glycomimetic compounds. Glycated LDL (g-LDL; 10 µg/mL) induced calcification of VSMCs after 21 days, compared to untreated control, which was inhibited by novel glycomimetic compounds C2 and C3 (1 µM), as shown by evaluation of markers of calcification. (**A**) Alizarin red staining (Bar = 100 µm) was eluted, (**B**) quantified and represented as fold change versus untreated control. (**C**) Alkaline phosphatase (ALP) activity at day 4. (**D**) VSMC protein lysates were collected after 48 h of treatment and levels of LDL receptor (LDL-R) determined. Data are mean ± SEM, *n* = 3–5 independent experiments. * *p* < 0.05, ** *p* < 0.01 and *** *p* < 0.001.

Based on the findings that CLI patients have elevated levels of glycation in their serum and the strong links between glycation and calcification [34,37], we next used the g-LDL model of induced calcification to establish whether glycomimetics may directly inhibit glycation-induced calcification. Firstly, we demonstrated a dose-response curve and identified a correlation between increasing doses of g-LDL, ranging from 10–30 mg/mL (Supplementary Figure S1) and increased calcification of VSMCs in vitro respectively (Supplementary Figure S1A), supported by AR quantification (Supplementary Figure S1B) and a measure of levels of glycation between BSA, g-BSA, LDL and g-LDL (Supplementary Figure S1C). For our comparative model, we used 10 µg/mL g-LDL for future experiments to stimulate osteogenic differentiation of human VSMCs, since this concentration induced calcification compared to untreated osteogenic controls (*p* < 0.01; Figure 3A,B) and was more physiologically relevant [38,39]. Once again, this effect was significantly attenuated by the glycomimetic compounds C2 and C3, as revealed by reduced AR staining at day 21 (*p* < 0.05; Figure 3A,B). As expected, g-LDL significantly increased ALP activity > 4 fold, while in the presence of both C2 and C3, ALP activity was significantly (*p* < 0.001 and *p* < 0.01, respectively) decreased at day 4 (Figure 3C). Of note, the g-LDL treatment increased LDL receptor levels after 48 h compared to the osteogenic control VSMCs (*p* < 0.05), which was attenuated by both C2 and C3 glycomimetics (*p* < 0.01; Figure 3D).

### 3.5. Glycomimetics Act via Inhibition of the RAGE/ERK/CREB Pathway

**Figure 4:**
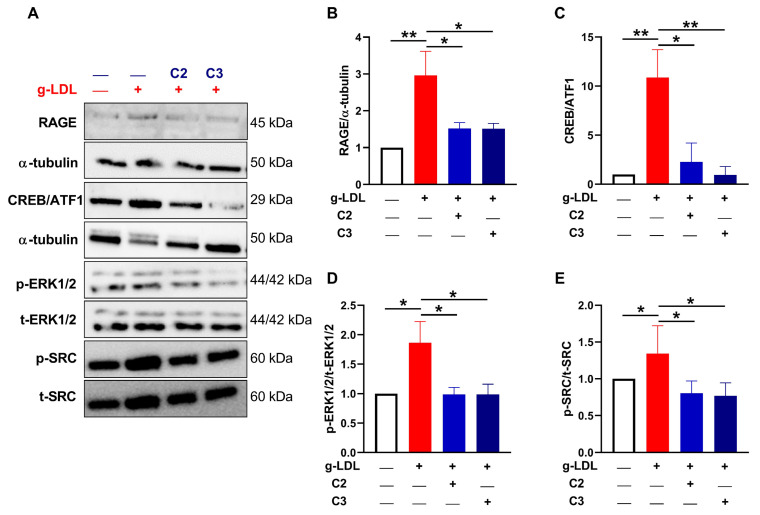
Modulation of protein phosphorylation and receptor levels by glycomimetics in a calcification model. VSMCs were stimulated with g-LDL (10 µg/mL), with and without novel glycomimetic compounds C2 and C3 (1 µM) and protein lysates were collected for (**A**) Western blot analysis at 48 h (RAGE), 24 h (pCREB/ATF-1) and 1 h (p-ERK/t-ERK and p-src/t-src ratios) and (**B**–**E**) quantified. All of these markers were increased by g-LDL compared with untreated (UN) control, which were attenuated by glycomimetics. Data are mean ± SEM, *n* = 3 independent experiments. * *p* < 0.05 and ** *p* < 0.01.

Given the findings that RAGE expression was localised within the vicinity of the calcified vessels from CLI patients, we next examined the levels of RAGE in our in vitro model of VSMC calcification. Incubating cells in the presence of g-LDL significantly increased the extent of RAGE expression at 48 hrs post-treatment, when compared to the untreated osteogenic controls, as shown by Western blot analysis (*p* < 0.01; Figure 4A,B). In addition, RAGE expression was significantly reduced by glycomimetics C2 and C3 (*p* < 0.05, Figure 4A,B).

To identify potential pathways downstream of RAGE activation, and subsequent mineralisation, a human phospho-kinase array and bioinformatic software were used to profile differentially active protein tyrosine kinases and to investigate the unique kinase networks activated by g-LDL-induced calcification and attenuated by the glycomimetics C2 and C3. Of the 27 proteins tested in the phosphor array, four were found to be significantly affected by the small molecular glycomimetics, namely the non-receptor tyrosine kinase mTOR (mammalian target of rapamycin), a member of the PI3-kinase-related kinase (PIKK) family [40,41]; the cyclic AMP (cAMP) response element binding protein (CREB); the serine/threonine protein kinase, Lyn (Lck/yes-related novel tyrosine kinase [42,43] and Yes (the cellular homolog of the oncogenic protein encoded by the Yamaguchi Sarcoma Viral Oncogene Homolog 1 and Esh avian sarcoma virus) [44], the latter two belong to the src family (Supplementary Figure S2). These four proteins demonstrated elevated phosphorylation following the addition of g-LDL to VSMCs incubated in osteogenic media, an effect that was attenuated in the presence of glycomimetics C2 and C3. These findings were then further validated by Western blotting.

We confirmed that one of the identified proteins, namely CREB, which was previously shown to be linked to calcification [45] demonstrated a significant increase in CREB/ATF1 phosphorylation after g-LDL stimulation (*p* < 0.01), which was reduced after glycomimetic treatment (Figure 4A,C). Next, to identify the links between the RAGE activation and the downstream CREB signalling pathway, we investigated the activation of extracellular signal-regulated kinase ERK, since it is known to be downstream of RAGE [46,47] and upstream of CREB [48]. The phosphorylated ERK/total-ERK ratio was increased by g-LDL treatment (*p* < 0.05) compared with the untreated control and significantly reduced after glycomimetic treatment (*p* < 0.05; Figure 4A,D).

Since it is known that src is activated downstream of RAGE [49], we next investigated the regulation of src, which was significantly elevated after g-LDL treatment (*p* < 0.05; Figure 4A,E) and, again, significantly decreased after glycomimetic treatment (*p* < 0.05; Figure 4A,E).

**Figure 5:**
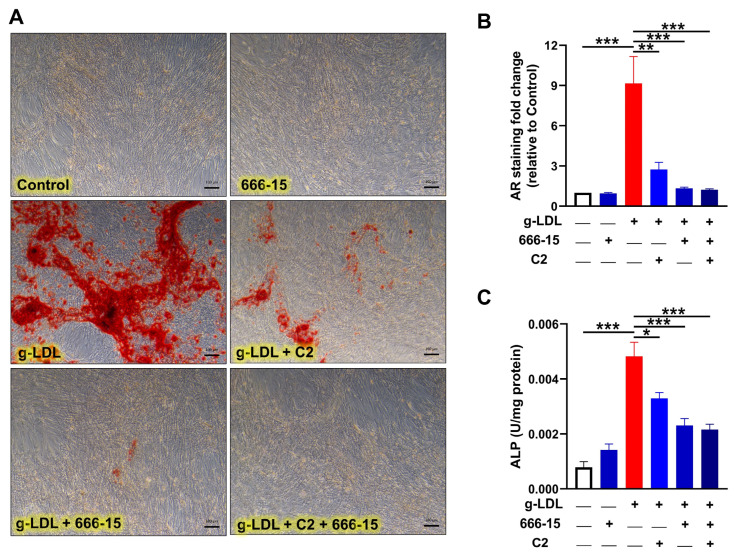
Inhibition of glycated LDL (g-LDL)-induced calcification by the CREB inhibitor, 666-15. VSMCs were treated with g-LDL (10 µg/mL), with and without the novel glycomimetic compound; C2 (1 µM), in the presence or absence of 666-15 (CREB inhibitor; 1 µM). (**A**) On day 21, VSMCs were stained with alizarin red (scale bar = 100 µm), which was eluted, (**B**) quantified and represented as fold change versus untreated control. g-LDL-induced calcification compared to untreated controls and was attenuated by glycomimetic C2, with a further reduction by 666-15. (**C**) C2 and/or 666-15 decreased ALP activity in g-LDL-induced VSMCs. Data are mean ± SEM, *n* = 3 independent experiments. * *p* < 0.05, ** *p* < 0.01 and *** *p* < 0.001.

Next, to confirm CREB involvement in our model of calcification and the anti-calcification effects of glycomimetic C2, we incubated cells in osteogenic media, with and without the addition of g-LDL, alongside cells also treated with the pharmacological inhibitor of CREB, (666-15) [50,51]. Here, we focussed on C2 only since it exhibited a trend towards a more enhanced effect than C3 in the previous experiments. Both AR staining (Figure 5A) and its quantification (Figure 5B) clearly show the calcification inhibitory effects of blocking CREB signalling with 666-15 and also confirm that g-LDL-induced calcification was significantly attenuated by C2 (*p* < 0.01), supported by the significant decrease in ALP activity in g-LDL-induced cells treated with 666-15 and/or C2 (Figure 5C). These findings add support to previously published work revealing CREB as an essential transcription factor in the calcification process [45]. In summary, elevated AGEs and g-LDL, which are elevated in CLI patient serum contribute to vascular calcification, and the SMC osteogenic potential is modulated by novel glycomimetic compounds, via the RAGE/src/ERK/CREB signalling cascade.

### 3.6. In Silico Binding of Glycomimetics with RAGE V Domain

**Figure 6:**
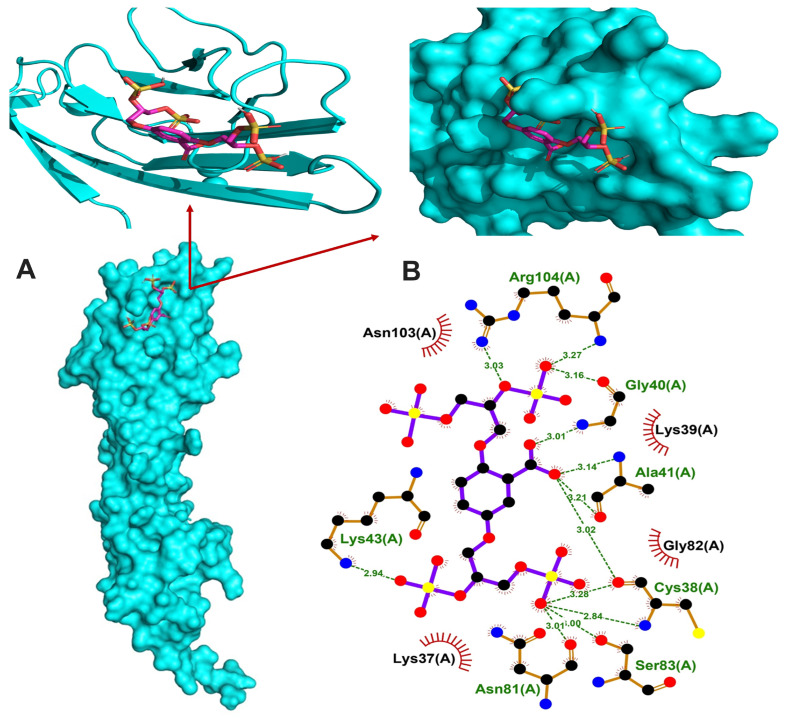
The binding model of RAGE V domain and C2. (**A**) Co-crystallized structure of RAGE VC1 domain with bound glycomimetic C2 and expanded pictures showing the binding region. (**B**) Representation of the intermolecular interactions between C2 and RAGE V domain binding site.

**Figure 7:**
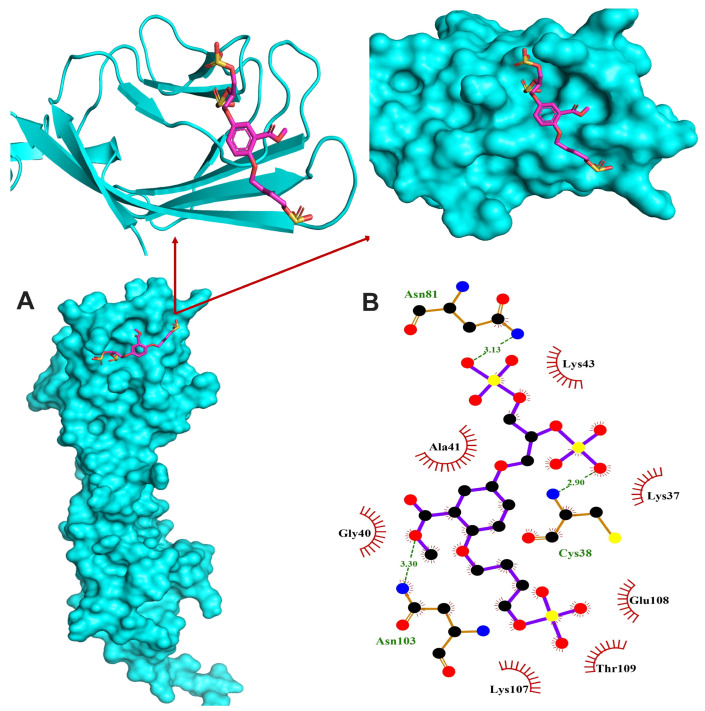
The binding model of RAGE V domain and C3. (**A**) Co-crystallized structure of RAGE VC1 domain with bound glycomimetic C3 and expanded pictures showing the binding region. (**B**) Representation of the intermolecular interactions between C3 and RAGE V domain binding site.

In order to investigate whether C2 and C3 glycomimetics are able to bind the V domain of RAGE protein, we carried out in silico molecular docking simulations. Both glycomimetics showed binding affinities towards the V domain of RAGE and the reported lowest binding energies were −5.7 and −5.4 kcal/mol for C2 and C3, respectively. The RAGE inhibitor TTP488 bound to the V domain with the lowest binding energy of −5.9 kcal/mol and exhibited hydrogen bonding with 2 amino residues and hydrophobic interactions with 7 residues (Supplementary Table S2 and Supplementary Figure S3). C2 showed hydrogen bonding with 7 amino acid residues and hydrophobic interactions with 4 residues, as shown in Figure 6, whereas C3 exhibited hydrogen bonding with 3 residues and hydrophobic interactions with 7 amino acid residues (Figure 7). Given their structural similarity, both C2 and C3 exhibited hydrogen bonding with the residues Cys38, and Asn81, and hydrophobic interaction with the residue Lys37.

## 4. Discussion

This study provides new evidence that g-LDL activates pathways that directly enhance matrix mineralisation by VSMCs, an effect that can be attenuated by the addition of glycomimetics. These findings support the potential therapeutic value of glycomimetic compounds to reduce the progression of vascular calcification. Glycomimetics are a new class of small-molecule selective drugs that address the disadvantages of previous carbohydrate leads, namely their low activity and insufficient drug-like properties [52].

The carbohydrate coating on all cell surfaces, known as the glycocalyx, plays a key role in cellular communication, through the stabilisation of ligand–receptor interactions via specific conformational changes in its structure [53,54]. The C2 and C3 glycomimetics used in this study, which were synthesised as small molecule HS mimics, have the potential to target aberrant signalling and protein–protein interactions, and provide the basis for innovative therapeutic strategies to address current unmet needs in cardiovascular disease [55]. The potential of GAGs and proteoglycans to regulate biological processes, in particular, their role in modulating cellular signalling events, makes them exceptionally appealing as drug candidates for multiple disease conditions [56]. Despite the fact that vascular calcification is a sinister pathology related to the increased risk of cardiovascular events [57], it has limited therapeutic management and represents an unmet clinical need that should be urgently addressed.

Our previous studies have revealed the important protective effects of glycomimetics on endothelial cells, where we demonstrate (i) a reduction in oxidative stress via Akt/eNOS and Nrf2/ARE signalling in HUVECs [20]; (ii) a correlation of impaired cell migration with structural alterations of HSPGs in an in vitro model of aging cells [18]; and (iii) improvement in the angiogenic repair capacity of endothelial colony-forming progenitor cells following treatment with these glycomimetics [19]. In light of this and our current findings, we propose that as well as acting directly on VSMCs, the endothelium could be the first line of defence against atherosclerosis and calcification, when treated with glycomimetics. In addition, we have also previously shown that oxidised LDL accelerates human VSMC mineralisation in vitro [58] and there is evidence to suggest that AGEs play a pathogenic role in calcification [59,60], although data utilising human cells is limited and the mechanistic action remains unknown. Here, we hypothesised that glycomimetics may disrupt g-LDL-RAGE interaction and prevent the aberrant osteoblastic differentiation and calcification of human VSMCs.

In the present study, using immunolocalisation studies, we demonstrate the presence of RAGE in highly calcified vessels from patients undergoing surgery for CLI, and also demonstrate elevated levels of glycation and bone markers (OPG and SOST) in the same patient serum. It was of interest that the level of the bone-related protein OCN was lower in serum from the CLI patients compared to the control, which correlates with the work of Ducy et al. showing that OCN knockout mice have increased bone formation [61]. However, we have previously shown OCN localisation in calcified vascular tissue [62] with a positive correlation to the extent of vascular calcification in humans, so it would appear there is a disparity between circulating concentrations of OCN and its role in tissue calcification [63]. It has been suggested that OCN is a hormone orchestrating the response to danger [64] and, in our study, the decreased OCN in the serum of CLI patients compared to healthy controls may reflect the patient’s inability to protect itself against the deposition of a mineralised matrix [65]. Furthermore, it has been established that bone is regulated by circadian rhythms, and, amongst the proteins involved in bone metabolism that oscillate over a 24 h period, OCN [66] was found in higher concentrations in the morning compared to the afternoon [67]. Of note, surgical amputation of the CLI patients recruited in this study was scheduled in the later part of the day. Therefore, the timing of the harvesting of blood in the evening offers further evidence that the levels of OCN follow a circadian rhythm, with low levels of this molecule present in the circulation in the evening [68,69], a finding that warrants further study, as the timing of surgery could impact the outcome for patients.

It is also of interest that in the in vitro model used in this study, VSMCs, when treated with patient serum, showed increased OCN secretion alongside calcification, which supports the findings that it is a bone-related marker [70]. Here, VSMCs are in direct contact with numerous factors and proteins present in the patient’s serum, and in combination, may amplify vascular calcification via Runx2 [62] or other signalling pathways, leading to increased OCN secretion. This is supported by our observation that VSMC calcification was evident earlier in serum-treated VSMCs at day 10 compared to our standard β-GP-induced calcification of 14–21 days [22,23].

Using our well-established in vitro model of vascular calcification, we show the acceleration of osteogenic differentiation of VSMCs treated with CLI serum compared to healthy serum and also increasing calcification with an increasing dose of g-LDL, suggesting that the elevated glycation and AGEs present in the CLI serum may be contributing to the calcification process. Furthermore, we also demonstrate that the novel HS mimics C2 and C3, were able to attenuate both g-LDL- and CLI patient serum-induced calcification of VSMCs. This finding was confirmed by a reduction in AR staining, CLI serum-induced ALP activity and OCN secretion in VSMCs incubated with C2 and C3 glycomimetics in vitro. Of note, Western blot analysis of lysates harvested from VSMCs treated with g-LDL in the presence or absence of C2 and C3 demonstrated that g-LDL induced both LDL receptor and RAGE protein expression, an effect which was inhibited in the presence of glycomimetics C2 and C3. These data strongly suggest that the glycomimetics have a protective effect in modulating the osteogenic inductive capacity of g-LDL, adding strength to our previous findings that our aryl-templated, small molecule HS-glycomimetics attenuate vascular calcification [21].

Since we show that the presence of C2 and C3 antagonises RAGE and LDL receptor signalling and it has been established that HS plays a key role in cell signalling in the vasculature via high mobility group protein B1, which is the major target for RAGE, and since this interaction further induces the downstream phosphorylation of ERK1/2 and p38 [13,71,72,73], we hypothesised that they interfere with downstream kinase signal transduction events that lead to osteogenic differentiation of VSMCs.

In order to identify a mechanism of action for the glycomimetics, and to probe the intracellular signalling pathways, 27 proteins were tested in a phosphor array, with four being found to demonstrate elevated phosphorylation following the addition of g-LDL to VSMCs incubated in osteogenic media, an effect which was not apparent in the presence of the small molecular glycomimetics. Consistent with previously described reports, showing LYN involvement in disease [74,75], the resultant Peptide-based kinase array profiling identified activation of LYN following g-LDL treatment of VSMCs, and reduced signalling following treatment with C2 and C3. Another protein identified was Yes, the most widely expressed member of the Src family of nonreceptor tyrosine kinases, which also regulates an array of cellular processes, including growth factor signalling, cytoskeletal dynamics, and cell proliferation [75]. After a 24 h incubation of VSMCs in g-LDL in the presence or absence of C2 and C3, we show reduced phosphorylation of another src protein mTOR, as well as the transcription factor CREB. Furthermore, we corroborated the involvement of CREB in the calcification process, using pharmacological inhibition of the CREB signalling pathway by 666-15 to prevent calcification, thus supporting previously published work that CREB is an essential transcription factor in the calcification process [45].

It has been suggested that in peripheral arterial disease and subsequent CLI, dysfunction of the cells of the vessel wall may be in part the result of their interaction with glycated proteins, such as g-LDL, supported by the findings of Wang et al. [76] who showed that an AGE compound, carboxymethyl lysine (CML), accelerates calcification in diabetes. Protein glycation occurs as a consequence of the non-enzymatic glycosylation of the free amino groups of lysine residues of apoB [77,78], and it appears that LDL is susceptible to glycation due to the concurrent reaction with elevated levels of free radicals present under atherosclerotic conditions [12].

We next demonstrated that g-LDL increased the p-src/t-src and p-ERK/t-ERK ratios and increased the phosphorylation of CREB/ATF-1. All of these events were attenuated by the novel glycomimetics C2 and C3. Src kinase plays an important role in RAGE-mediated inflammatory gene expression and cell migration, key events associated with diabetic and CLI vascular complications [49,75]. Taken together, our data support and extend the findings of others, where src is targeted by activated RAGE, an immunoglobulin superfamily member, and could be implicated in the development of calcification. In addition, the signal transduction activating ERK has previously been shown to be activated during the osteoblastic differentiation and mineralisation of calcifying vascular cells in vitro [79], data which are further supported by our current findings, where we show that ERK was activated by g-LDL, an effect that could be reversed in the presence of glycomimetics. Furthermore, activation of the CREB signalling cascade via phosphorylation has been shown to mediate a number of mechanisms in VSMCs, including proliferation [80] and cell migration [81], processes that play key roles in atherosclerosis and vascular calcification. CREB is also a recognised transcriptional regulator of a number of inducers of calcification, among which is OCN [82]. Therefore, we interrogated the ATF/CREB signalling pathway to establish whether it may be involved in g-LDL or AGE-induced osteogenic differentiation of VSMCs in vitro.

To further explore the mechanism underlying the modulating effect of glycomimetics on RAGE signalling, we investigated the binding affinity toward the V domain of RAGE in silico. RAGE is a 45 kDa protein that consists of extracellular, transmembrane and cytoplasmic domains. The extracellular structure is composed of the V domain and C1 domain connected by a flexible amino acid linker to a C2 domain [83]. Given that RAGE-mediated inflammation, ROS generation and vascular calcification [5,6,7,8] are elicited via ligand binding, the best approach to attenuate these pathologic processes is to inhibit ligand binding. AGEs recognise the V domain of RAGE through a specific binding pattern [30] and the majority of RAGE activators bind this domain [84]. Therefore, the V domain is of paramount importance for the development of RAGE inhibitors. Interestingly, both of our glycomimetics showed binding affinities toward the V domain with comparable binding energy to that of the established inhibitor TTP488. The stability of the glycomimetics–RAGE complexes was further demonstrated by hydrogen bonding and hydrophobic interactions with various amino acid residues.

**Figure 8:**
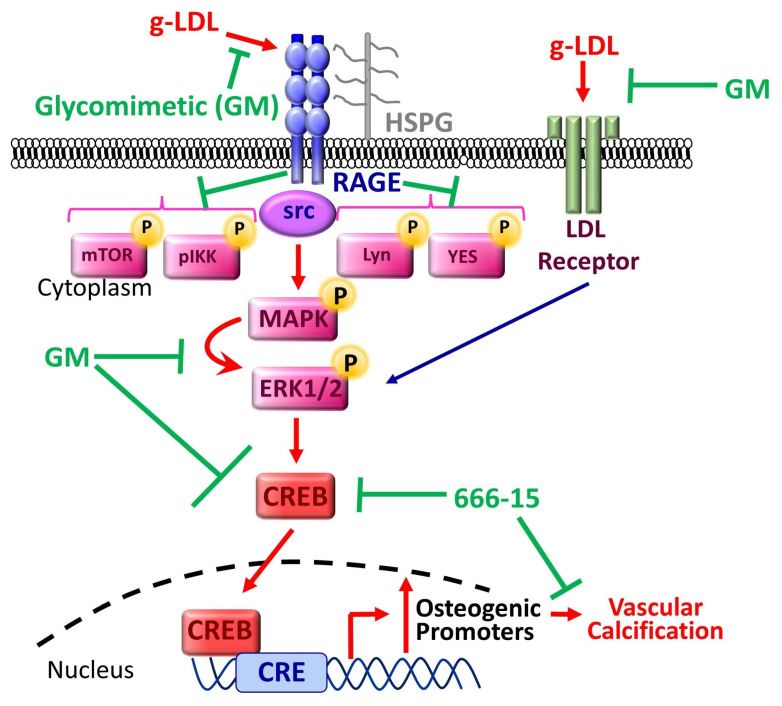
Schematic diagram highlighting the proposed inhibition of vascular calcification by our novel glycomimetic compounds. Glycomimetics operate via heparan sulphate proteoglycans (HSPG) and respective receptors, e.g., RAGE or LDL receptors, and activate downstream signalling via various phosphor kinases as shown. These in turn activate CREB which translocates to the nucleus and through binding to a range of promoter elements causes transcription of osteogenic factors resulting in increased mineralisation of VSMCs and calcification within the tissue. g-LDL—glycated low-density lipoprotein; RAGE—receptor for advanced glycation end products; HSPG—heparan sulphate proteoglycans; LDL—low-density lipoprotein; MAPK—mitogen-activated protein kinase; ERK—extracellular signal-regulated kinase; CREB—cyclic AMP response element-binding protein.

In summary, elevated AGEs and g-LDL, which are elevated in CLI patient serum, contribute to vascular calcification, and the SMC osteogenic potential is modulated by novel glycomimetic compounds, via the RAGE/src/ERK/CREB signalling cascade, as summarised schematically in Figure 8.

## 5. Conclusions

This study extends the findings regarding the protective properties of glycomimetics compounds, showing that small molecule glycomimetics, exemplified by compounds C2 and C3, have promising protective effects against downstream vascular calcification amongst other important roles in in vitro and ex vivo studies [52]. Therefore, in agreement with Stabley & Towler, 2017 [85], the ability to limit the deposition of a calcified matrix, targeted at lowering AGE levels and RAGE expression, could translate into clinical practice and improve the quality-of-life of this patient group. Based on these and other findings, we support the need to develop therapeutic strategies that may target specific PGs, and interfere with key pathways that promote vascular disease, thus improving the armamentarium for prevention of calcification. Although therapeutic options for diabetic patients with peripheral vascular disease are limited, in particular those with diffuse arterial calcification, the current findings offer novel prospects for this clinical group. However, with vascular calcification being such a complicated disease, a systems biology and modelling approach is warranted to elucidate and understand this multifactorial process more fully.

## Data Availability

Data will be available from the corresponding author on request.

## Supplementary Materials

The following supporting information can be downloaded at: https://www.mdpi.com/article/10.3390/cells13040312/s1, Figure S1: (A) Modulation of glycated-LDL (g-LDL) induced calcification is dose dependent and is greater than LDL-induced VSMC calcification. Alizarin red staining images, scale bar 100 µm. (*p* < 0.01) *n* = 3. (B) Quantification of alizarin red stain at 414 nm, represented as fold change vs. untreated. (C) Quantification of protein modification by glycation. A TNBSA assay was performed to quantify the extent of glycation. The TNBSA assay determines the extent of protein modification by determining free amino acid groups. Glycated forms of BSA and LDL (incubated with 50 mM methylglyoxal for 7 days at 37 °C) had a lower absorbance than BSA and LDL, respectively, this relationship was linear across a range of concentrations, indicating the extent of glycation. Figure S2: Modulation of protein kinase phosphorylation by glycomimetics using a human phospho-kinase array. SMCs were induced with glycated LDL and novel glycomimetic compounds (1 µM) over 24 h and protein isolated. Phosphorylation of CREB, TOR and the SRC proteins LYN, YES and CHK-2 were reduced with glycomimetics, whereas B-catenin, STAT 5 and C-JUN were increased. Figure S3: The binding model of RAGE V domain and TTP488. (A) Co-crystallized structure of RAGE VC1 domain with bound TTP488 and expanded pictures showing the binding region. (B) Representation of the intermolecular interactions between TTP488 and RAGE V domain binding site. Table S1: A list of primary antibodies used in this study. Rb—rabbit and Ms—mouse. Table S2: Lowest binding energy, hydrogen bonding and hydrophobic interactions of C2, C3 and TTP488 with the V domain of RAGE.
