# Supplementary material for: Novel Glycomimetics Protect against Glycated Low-Density Lipoprotein-Induced Vascular Calcification In Vitro via Attenuation of the RAGE/ERK/CREB Pathway
Source: Cells. 2024 Feb 8;13(4):312. doi: 10.3390/cells13040312 (PMC10887290; doi:10.3390/cells13040312)
Supplement: Supplementary file 1 [file cells-13-00312-s001.zip › cells-2825038-supplementary.pdf]

Supplementary Data

Article

Novel Glycomimetics Protect against Glycated Low-Density Lipoprotein-Induced Vascular Calcification In Vitro via Attenuation of the RAGE/ERK/CREB Pathway

Gary P. Sidgwick [1], Ria Weston [1], Ayman M. Mahmoud [1], Andrew Schiro [2,3], Ferdinand Serracino-Inglott [1,2,3], Shikha M.Tandel [1], Sarah Skeoch [4,5,6], Ian N. Bruce [4,5], Alan M. Jones [1,7], M. Yvonne Alexander [1] and Fiona L. Wilkinson [1,*]

[1] Department of Life Sciences, Manchester Metropolitan University, Manchester M1 5GD, UK; r.weston@mmu.ac.uk (R.W.); a.mahmoud@mmu.ac.uk (A.M.M.); ferdinand.serracino-inglott@mft.nhs.uk (F.S.-I.); standel01@qub.ac.uk (S.M.T.); a.m.jones.2@bham.ac.uk (A.M.J.); y.alexander@mmu.ac.uk (M.Y.A.)
[2] Cardiovascular Research Institute, University of Manchester, Manchester M13 9PL, UK; druschiro@hotmail.com
[3] Vascular Unit, Manchester University Hospitals NHS Foundation Trust, Manchester M13 9WL, UK
[4] Centre for Epidemiology Versus Arthritis, University of Manchester, Manchester M13 9PL, UK; sarah.skeoch1@nhs.net (S.S.); ian.bruce@manchester.ac.uk (I.N.B.)
[5] National Institute for Health Research Manchester Biomedical Research Centre, Manchester University Hospitals NHS Foundation Trust, Manchester Academic Health Science Centre, Manchester M13 9PL, UK
[6] Royal National Hospital for Rheumatic Diseases, Bath BA1 1RL, UK
[7] School of Pharmacy, University of Birmingham, Birmingham B15 2TT, UK
* Correspondence: f.wilkinson@mmu.ac.uk

Supplementary Table S1. A list of primary antibodies used in this study. Rb- rabbit and Ms-mouse.

Antibody Target (Species)	Company (catalogue ID)	Dilution
p-ERK (Rb)	R+D Bio-Techne (MAB1018)	1:1000
t-ERK (Ms)	R+D Bio-Techne (MAB1576)	1:1000
p-AKT (Rb)	R+D Bio-Techne (AF887)	1:400
t-AKT (Ms)	R+D Bio-Techne (MAB2055)	1:2500
p-CREB (ms)	BD Pharmingen (558359)	1:2000
RAGE (Rb)	Abcam (ab3611)	1:1000
LDL Receptor (Rb)	Abcam (ab52818)	1:1000
p-CMET (Rb)	Abcam (ab5662)	1:1000
t-CMET (Rb)	Abcam (ab51067)	1:1000
B-catenin Ms)	BD Pharmingen (610154)	1:2000
a-Tubulin (Ms)	Abcam (ab7291)	1:5000

Supplementary Table S2. Lowest binding energy, hydrogen bonding and hydrophobic interactions of C2, C3 and TTP488 with the V domain of RAGE.

	**Lowest binding energy (kcal/mol)**	**Hydrogen bonding**	**Hydrophobic interactions**
**C2**	-5.7	Arg104, Gly40, Ala41, Cys38, Ser83, Asn81, and Lys43	Asn103, Lys37, Lys39, and Gly82
**C3**	-5.4	Cys38, Asn103, and Asn81	Lys43, Lys37, Glu108, Thr109, Lys107, Gly40, and Ala41
**TTP488**	-5.9	Glu94 and Arg114	Ala152, Pro151, leu214, Gly95, Gly56, Tyr150, and Phe186

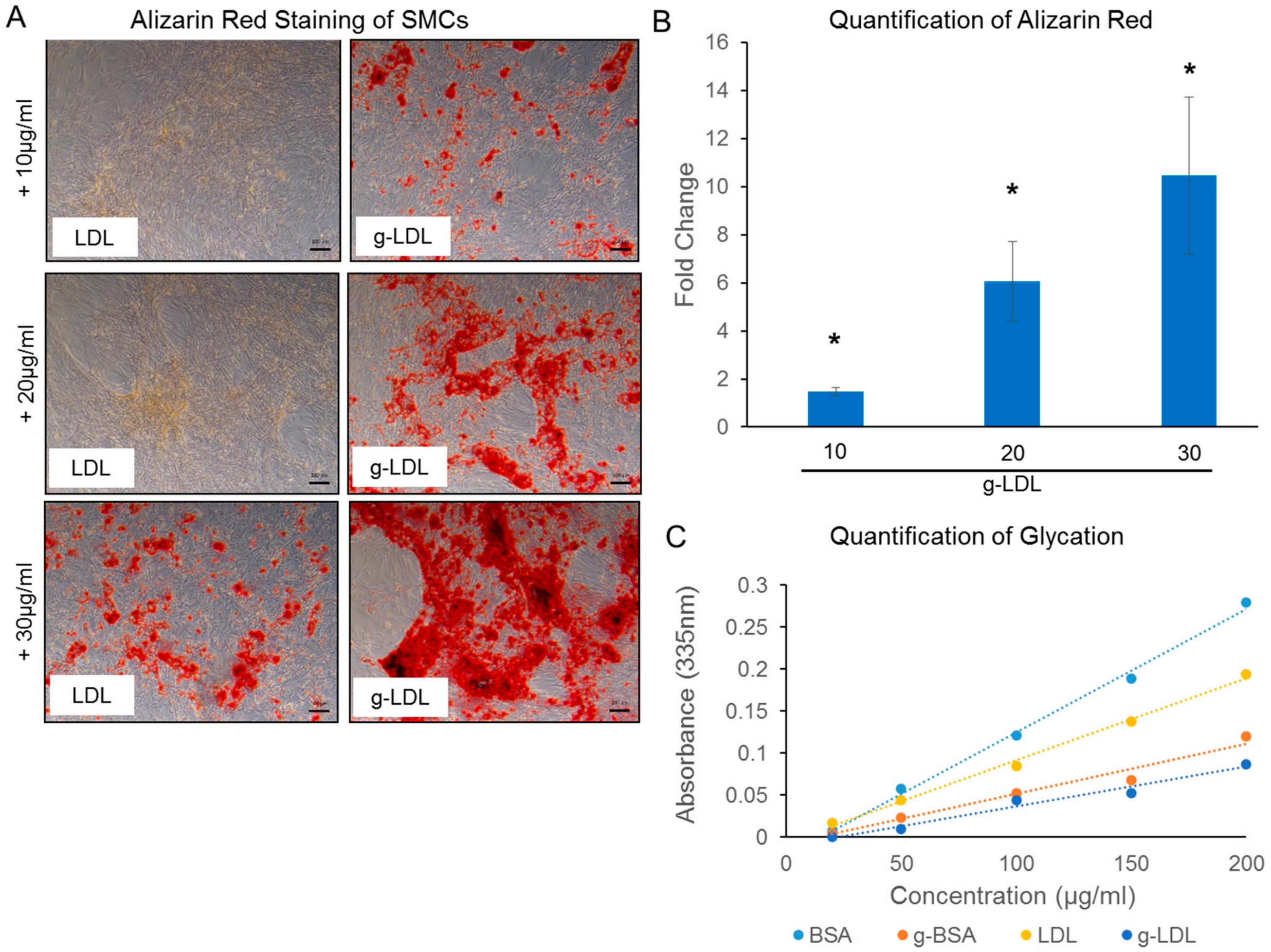

Supplementary Figure S1. (A) Modulation of glycated- LDL (g-LDL) induced calcification is dose dependant and is greater than LDL–induced VSMC calcification. Alizarin red staining images, scale bar 100µm. ($p<0.01$) n=3. **(B)** Quantification of alizarin red stain at 414nm, represented as fold change vs untreated. **(C)** Quantification of protein modification by glycation. A TNBSA assay was performed to quantify the extent of glycation. The TNBSA assay determines the extent of protein modification by determining free amino acid groups. Glycated forms of BSA and LDL (incubated with 50mM methylglyoxal for 7 days at 37°C) had a lower absorbance than BSA and LDL respectively, this relationship was linear across a range of concentrations, indicating the extent of glycation.

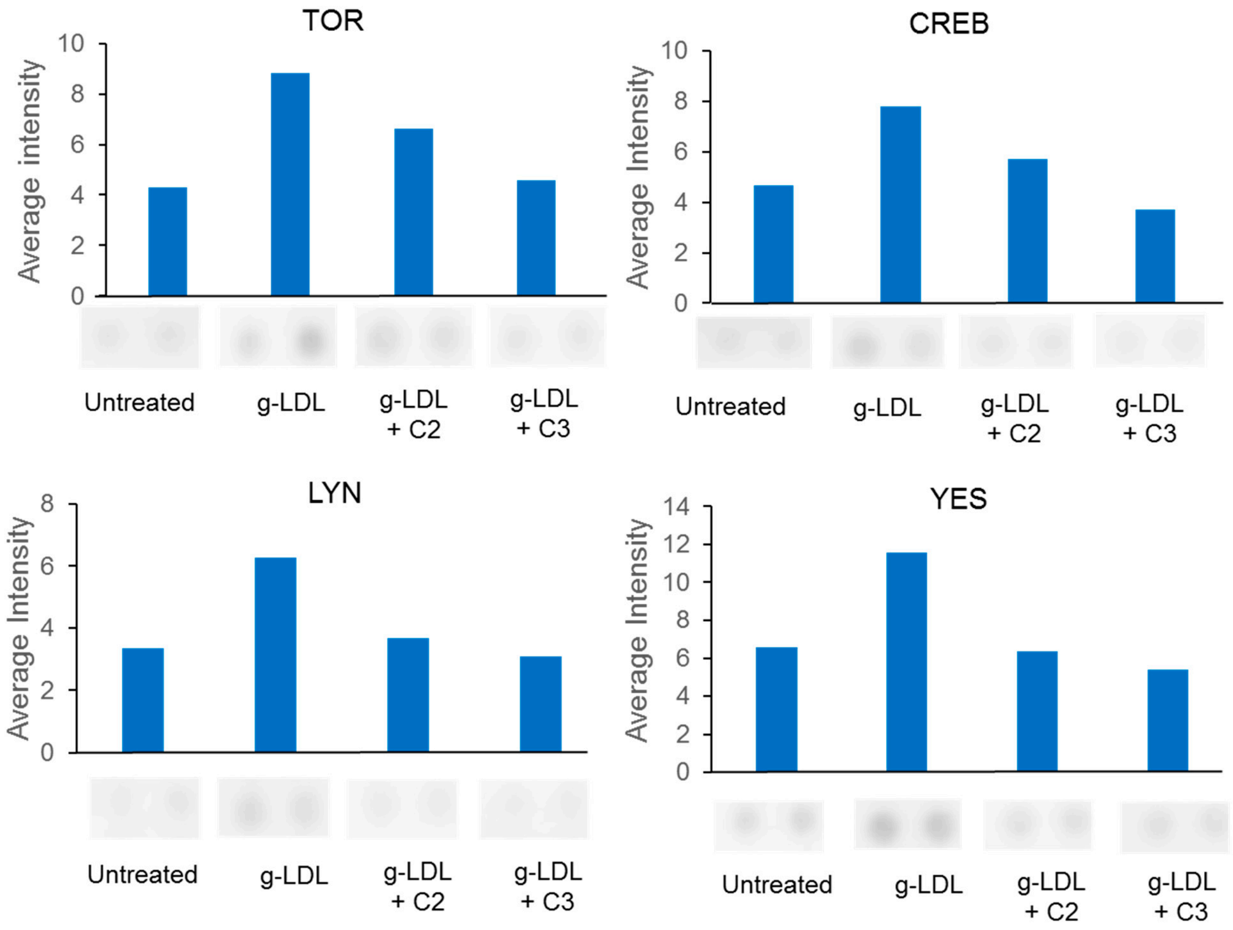

Supplementary Figure S2. Modulation of protein kinase phosphorylation by glycomimetics using a human phospho-kinase array. SMCs were induced with glycated LDL and novel glycomimetic compounds (1μM) over 24hrs and protein isolated. Phosphorylation of CREB, TOR and the SRC proteins LYN, YES and CHK-2 were reduced with glycomimetics, whereas B-catenin, STAT 5 and C-JUN were increased.

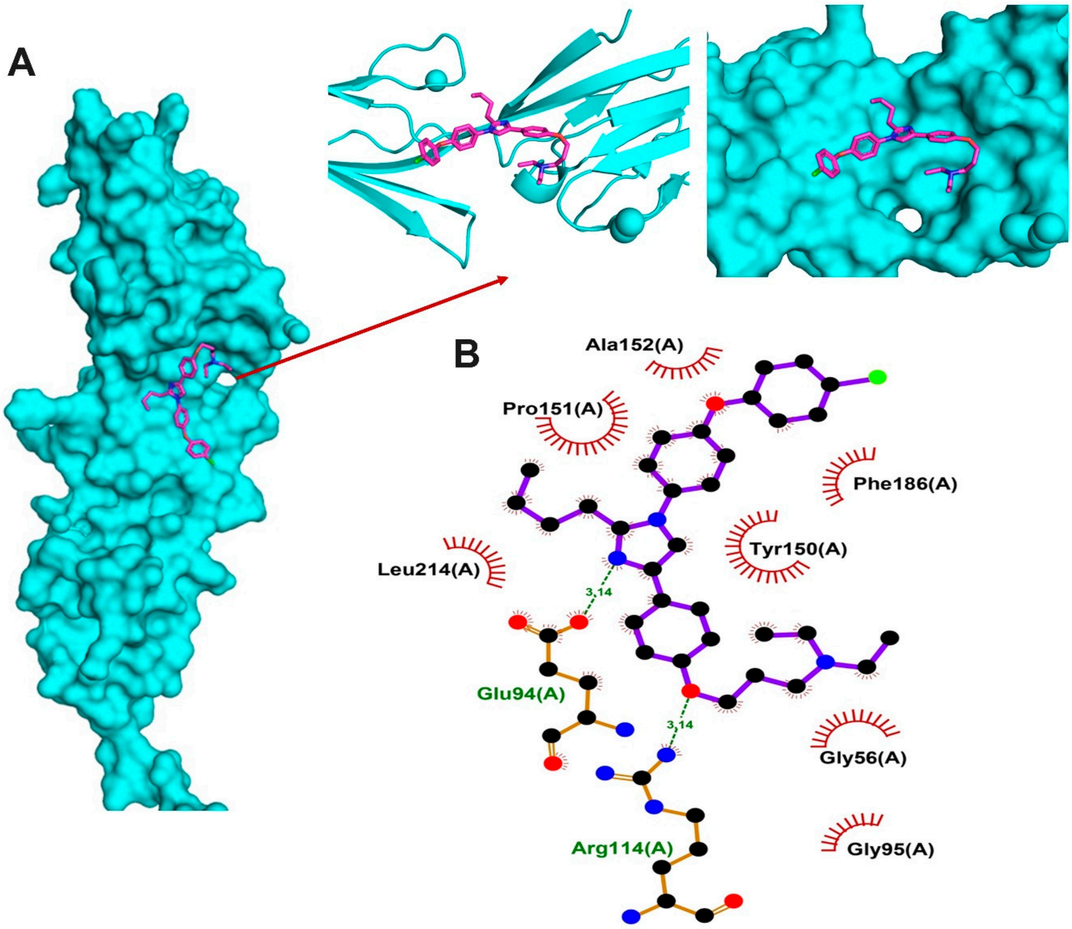

Supplementary Figure S3. The binding model of RAGE V domain and TTP488. (**A**) Co-crystallized structure of RAGE VC1 domain with bound TTP488 and expanded pictures showing the binding region. (**B**) Representation of the intermolecular interactions between TTP488 and RAGE V domain binding site.
